# UWB Bandpass Filter with Dual Notched Bands Using T-Shaped Resonator and L-Shaped Defected Microstrip Structure

**DOI:** 10.3390/mi9060280

**Published:** 2018-06-01

**Authors:** Xuemei Zheng, Yuwen Pan, Tao Jiang

**Affiliations:** 1 College of Information and Communication Engineering, Harbin Engineering University, Harbin 150001, China; zhengxuemei@hrbeu.edu.cn; 2 College of Information Engineering, Northeast Electric Power University, Jilin 132012, China; 3 Research and Development Department, Sainty-Tech Communications Limited, Nanjing 211100, China; peter.pan@sainty-tech.com

**Keywords:** microstrip filters, defected microstrip structure, microstrip circuits, bandpass filter

## Abstract

In this paper, an ultra-wideband (UWB) bandpass filter (BPF) with dual notched bands using a T-shaped resonator and L-shaped defected microstrip structure (DMS) is proposed and fabricated. First, the principle of generating notched bands by using a T-shaped resonator and L-shaped defected microstrip structure to determine the size parameters of the structure is analyzed. High frequency structure simulator (HFSS) software is used to analyze the performance of the filter, and advanced design system (ADS) is used to extract the equivalent circuit model parameters. The two simulation results are consistent, which further verifies the correctness of the circuit model. Finally, the filter is fabricated and measured. The measured results are in good agreement with simulated results, demonstrating good insertion loss and return loss. The proposed filter has dual independently controllable notched bands which are implemented by coupling the T-shaped resonator to the transmission line and by etching the L-shaped defected microstrip structure respectively. The proposed filter can suppress dispensable bands at 3.5 GHz and 7.5 GHz in WiMAX band and X-band to improve the performance of the ultra-wideband communication system. By adjusting the parameters of the T-shaped resonator and L-shaped defected microstrip structure, the UWB bandpass filter with dual notched bands working at WiMAX band and X-band can be designed and applied to the wireless communication system.

## 1. Introduction

Ultra-wideband (UWB) communication systems are widely used in wireless communication because of low cost and high data rate. The ultra-wideband (3.1–10.6 GHz) frequency spectrum was announced by the Federal Communications Commission in 2002 [1] for unlicensed indoor and hand-held commercial applications. ultra-wideband filters, as one of the key components of the radio frequency front-end, directly affect the quality of communication systems. Some other existing narrowband services already occupy frequencies in the ultra-wideband band, such as WiMAX in some Asian and European countries (3.4–3.6 GHz), and X-band satellite communication services frequency band (7.25–8.395 GHz). Therefore, it is necessary to design a ultra-wideband bandpass filter with notched bands. In order to realize the miniaturization and narrow-band notch of microwave integrated circuits, ultra-wideband filters require better frequency selection performance, a more compact structure, and seamless integration. 

A number of techniques to design ultra-wideband bandpass filters with notched bands have been studied [2,3,4,5,6,7,8,9,10,11,12,13,14,15,16]. The notch bands are generated by introducing a resonance unit, such as a stepped impedance resonator (SIR) [2,3] and multi-mode resonator (MMR) [4,5,6,7,8,9]. The disadvantage of adopting stepped impedance resonator or multi-mode resonator is that the size of the notch filter is too large and the transmission zero cannot be flexibly adjusted. The stop-band function is achieved by introducing defect ground structure (DGS) [10,11] and defect microstrip structure (DMS) [12,13]. Defect ground structure can effectively reduce the size of the filter, but it is difficult to obtain good broadband and sharp skirt selectivity. In addition, there is leakage of the floor. The microstrip filter based on defect microstrip structure can solve the floor leakage problem. The coplanar waveguide (CPW) [14,15,16] is used to achieve notch characteristics. The coupling between the microstrip line and the coplanar waveguide is strong and the filter structure is compact, but the stop band characteristic is generally not good. Novel dual-band bandpass filters are implemented for 3.5 GHz and 5.8 GHz in [17], and the pass band of the proposed filter covers 2.4 GHz, 2.5 GHz, and 3.5 GHz in [18]. In order to remove interference from WiMAX and satellite communication bands, a dual notched ultra-wideband bandpass filter is proposed in this paper.

A dual notched ultra-wideband bandpass filter using a T-shaped resonator and L-shaped defect microstrip structure is proposed and fabricated. The first and second notched band functions are respectively implemented by coupling the T-Shaped resonator to the transmission line and etching the L-shaped defect microstrip structure on the bandpass filter. In the second section, we will discuss the design of the ultra-wideband filter with dual notched bands and study the frequency rejection function based on a T-shaped resonator and L-shaped defect microstrip structure. In the third section, a dual bandpass filter with notched bands is fabricated and measured, with the measured results in good agreement with simulated results, demonstrating good insertion loss and return loss. By adjusting the parameters of the T-shaped resonator and L-shaped defect microstrip structure, the ultra-wideband bandpass filter with dual notched bands working at WiMAX band and X-band can be designed and applied to the wireless communication system.

## 2. Filter Design

**Figure 1:**
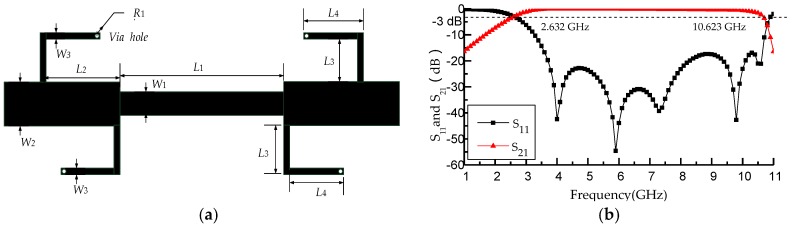
(**a**) Structure of ultra-wideband bandpass filter without T-shaped resonator and L-shaped defect microstrip structure; (**b**) simulation results of the ultra-wideband bandpass filter without T-shaped resonator and L-shaped defect microstrip structure.

The proposed dual notched ultra-wideband bandpass filter using a T-shaped resonator and L-shaped defect microstrip structure is designed on a bandpass filter in the literature [5]. The structure of a ultra-wideband bandpass filter without a T-shaped resonator and L-shaped defect microstrip structure is shown in Figure 1a. Figure 1b lays out the simulation results of a basic ultra-wideband bandpass filter by using high frequency structure simulator (HFSS) software (ANSYS, Inc., Canonsburg, PA, USA). To satisfy the requirements for ultra-wideband performance and improve edge steepness of the passband, four short-circuited stubs (length of λ/4) are used. Figure 1b indicates that the bandwidth of the bandpass filter is from 2.632 to 10.623 GHz and the fractional bandwidths (FBW) is 123.9%. The insertion loss of the ultra-wideband bandpass filter is less than 0.8 dB, while the simulated return loss is greater than 16 dB within the passband.

**Figure 2:**
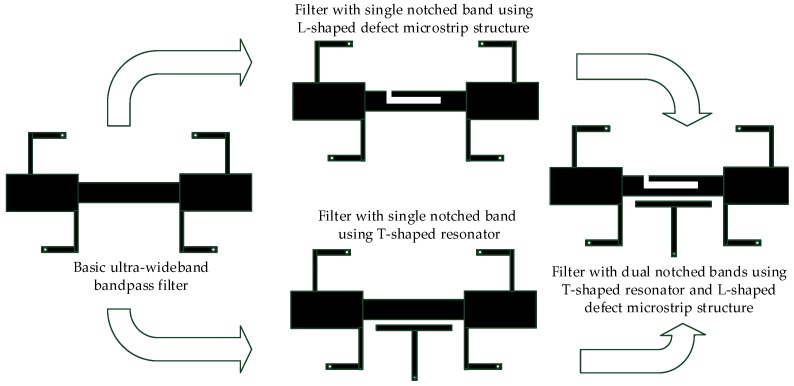
Design process of the dual notched bands filter.

**Figure 3:**
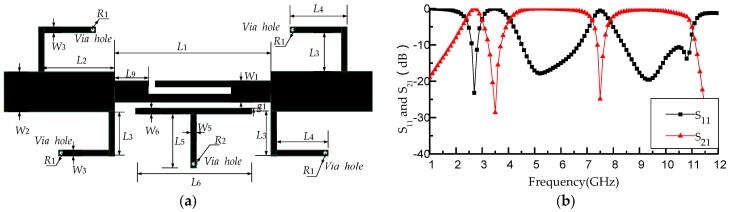
(**a**) The circuit of the proposed filter; (**b**) simulation results of the proposed filter.

Figure 2 shows the design process of the dual notched bands filter. The basic filter can form the first notched band by using the T-shaped resonator and the second notched band by using the L-shaped defect microstrip structure. Figure 3a,b shows the circuit configuration of the proposed filter and its S parameters, which are calculated by HFSS. The proposed filter achieves dual notched bands, whose center frequencies are at the 3.5 and 7.5 GHz, respectively. On notched frequencies, the corresponding reflection coefficient is −28.6 dB and −25.1 dB with a the fractional bandwidths (FBW) of 15.76% and 4.96%, respectively.

**Figure 4:**
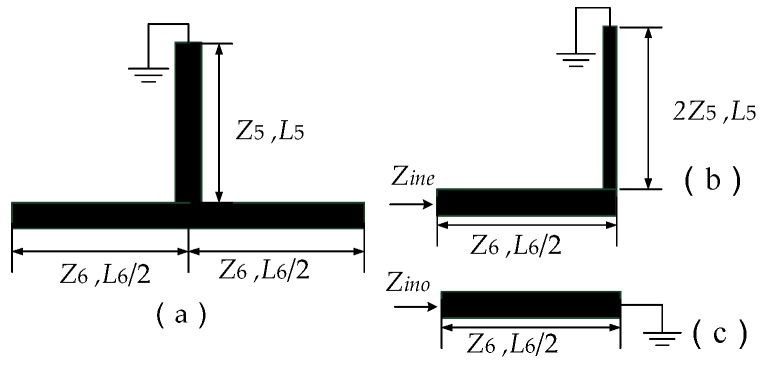
(**a**) The structure of T-shaped resonator; (**b**) even-mode equivalent circuit; (**c**) odd-mode equivalent circuit (*Z*_5_ and *Z*_6_ are the characteristic impedance. *L*_5_ and *L*_6_/2 are length of the open-circuited stub and the short-circuited stub).

Figure 4 shows the structure of the T-shaped resonator and further analyzes its even-mode and odd-mode equivalent circuit. By using odd-mode and even-mode analysis method [6], the input impedance of even-mode and odd-mode can be expressed as the Equations (1) and (2), where $\theta_{1}$ and $\theta_{2}$ are the electrical length of the open-circuited stub and the short-circuited stub, respectively.

(1) $$Z_{\mathrm{ine}}=jZ_{6}\frac{Z_{6}\tan\theta_{6}+2Z_{5}\tan\theta_{5}}{Z_{6}-2Z_{5}\tan\theta_{6}\tan\theta_{5}}$$

(2) $$Z_{\mathrm{ino}}=jZ_{6}\tan\theta_{6}$$

$\theta_{6}$ is defined as the electrical length of the open-circuited stub, $\theta_{5}$ is defined as the electrical length of the short-circuited stub, which can be expressed as:

(3) $$\theta_{5}=\beta L_{6},\;\;\;\theta_{6}=\beta L_{6}/2$$

The even-mode resonant frequency *f*_even_ and odd-mode resonant frequency *f*_odd_ can be expressed as shown in Equations (4) and (5). If the two signals are in the same phase, they are stacked together to form a pass band. However, in this frequency range, the two signals’ phase is opposite, and the stop band is formed. In order to further reduce the difficulty of design, the parameter is made *L*_6_ = 2*L*_5_. In this way, the resonant frequency of odd mode and even mode are only related to *L*_6_, and the first resonance frequency is adjusted by adjusting *L*_6_.

(4) $$f_{\mathrm{even}}=\frac{c}{4(L_{6}/2+L_{5})\sqrt{\varepsilon_{\mathrm{eff}}}}=\frac{c}{4(L_{6}/2+L_{6}/2)\sqrt{\varepsilon_{\mathrm{eff}}}}=\frac{c}{4L_{6}\sqrt{\varepsilon_{\mathrm{eff}}}}$$

(5) $$f_{\mathrm{odd}}=\frac{c}{2L_{6}\sqrt{\varepsilon_{\mathrm{eff}}}}$$

**Figure 5:**
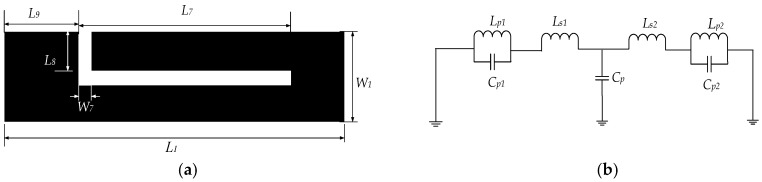
(**a**) The element structure of L-shaped defect microstrip structure; (**b**) the equivalent lumped circuit of T-shaped resonator and L-shaped defect microstrip structure.

Figure 5a illustrates the structure of the L-shaped defect microstrip structure, whose width is W_7_ and length is the sum of *L*_7_ and *L*_8_. The notch band characteristics are formed by using defect microstrip structure similar to using defect ground structure [19], and the resonant unit cell are modeled by L-shaped defect microstrip structure and the T-shaped resonator, i.e., Figure 4b, only considers the first and second resonant modes, which shows the equivalent lumped circuit of the resonant elements composed of the T-shaped resonator and L-shaped defect microstrip structure.

According to the theory of transmission line, each resonant element can be equivalent to a simple LC parallel resonant circuit with a characteristic that is similar to the first order Butterworth low-pass filter. Therefore, the equivalent circuit model Equations (6)–(10) of the two resonant elements composed of the T-shaped resonator and the L-shaped defect microstrip structure can be deduced from the Butterworth low-pass filter circuit model, where $f_{T}$ means a transit frequency, while $f_{01}$ and $f_{02}$ describe the lower and the higher resonant frequencies. The 3-dB bandwidth at $f_{01}$ and $f_{02}$ are denoted by $\Delta_{3\mathrm{dB}\_f_{01}}$ and $\Delta_{3\mathrm{dB}\_f_{02}}$. The imaginary parts of three Z parameters at $f_{T}$ are *X*_11_, *X*_22_ and *X*_21_. The following equivalent formulas regarding resonant elements composed of the T-shaped resonator and L-shaped defect microstrip structure can be derived from Reference [3]. The equivalent equations of resonant elements composed of the T-shaped resonator and L-shaped defect microstrip structure are shown in Reference [19].

(6) $$C_{pi}=\frac{1}{4\pi Z_{0}\Delta_{3\mathrm{dB}\_f_{0i}}}\mathrm{for}\;i=1,2$$

(7) $$L_{pi}=\frac{1}{{\left(2\pi f_{0i}\right)}^{2}C_{psi}}\mathrm{for}\;i=1,2$$

(8) $$C_{p}=\frac{1}{2\pi f_{T}X_{21}}$$

(9) $$L_{si}=\frac{X_{ii}-X_{21}}{2\pi f_{T}}+\frac{L_{pi}}{{\left(f_{T}/f_{0i}\right)}^{2}-1}\mathrm{for}\;i=1,2$$

**Figure 6:**
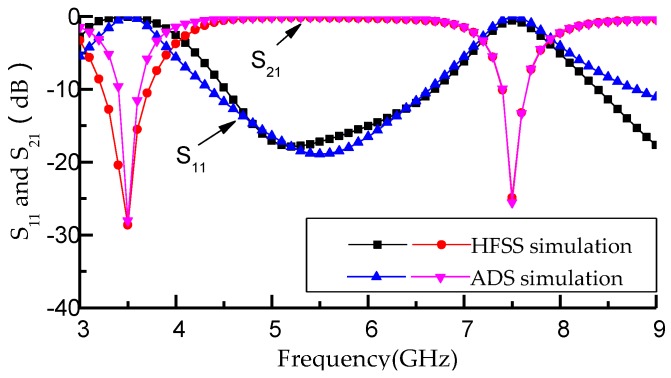
The comparison of simulation results by using high frequency structure simulator (HFSS) and advanced design system (ADS).

**Figure 7:**
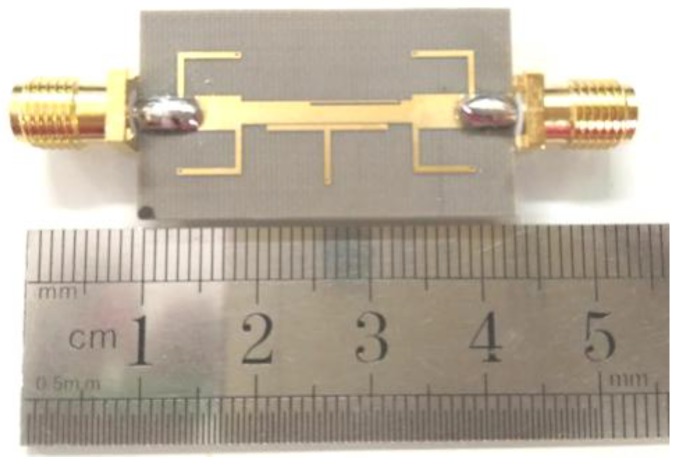
The photograph of the fabricated filter.

Defect microstrip structure has the same slow wave characteristics as DGS, so the resonant frequency can be derived from the method of DGS [20]. The L-shaped defect microstrip structure can be regarded as a half guided wavelength crooked slot line resonator. Therefore, the resonant frequency *f* may be achieved as:

(10) $$f=\frac{c}{2L\sqrt{\varepsilon_{\mathrm{slot}}}}$$

where *c* is the free-space speed of light, *L* = *L*_7_ + *L*_8_ the total length of the L-shaped defect microstrip structure, and $\varepsilon_{\mathrm{slot}}$ is the effective dielectric constant of the slot acquired by the closed-form equations in Reference [21]. The circuit parameters extracted from the ADS is shown in Table 1. Figure 6 shows the comparison of HFSS and ADS simulation results regarding the equivalent equation of resonant elements composed with the T-shaped resonator and L-shaped defect microstrip structure. It can be seen from Figure 6 that the ADS simulation results and the HFSS simulation results have the same resonant frequencies at 3.5 GHz and 7.5 GHz with the same insertion loss 28.6 dB, which proves the validity and correctness of the equivalent circuit. Figure 7 shows the photograph of the fabricated filter which selects RT/Duorid5880 (Rogers Corporation, Chandler, AZ, USA) as a substrate with the size of 32 mm × 10 mm × 1 mm.

## 3. Results and Discussion

In this paper, the T-shaped resonator is coupled to the basic ultra-wideband filter and the L-shaped defect microstrip structure is realized by etching an asymmetric L slot line whose width is *W*_7_. The T-shaped resonator is comprised of short-stub (*L*_5_, *W*_5_) and open-stub (*L*_6_, *W*_6_) with equal length and width (*L*_5_ = *L*_6_, *W*_5_ = *W*_6_) so that the impedance ratio (*K* = *Z*_2_/*Z*_1_) only depends on their electrical length. The final size of the filter is optimized by using HFSS with 0.1 GHz sweep step.

Optimized design parameters of the proposed ultra-wideband filter is given in Table 2. The simulated and fabricated results proved that the first notched band is adjustable by the parameters (*g*_1_, *L*_6_ and *W*_6_) of the T-shaped resonator, while the second notched band is controlled by the parameters (*L*_7_, *L*_8_ and *W*_7_) of the L-shaped defect microstrip structure.

The simulation results of the T-shaped resonator and L-shaped defect microstrip structure is given in Table 3 and Table 4. It can be concluded that the lower frequency notched band with a 3-dB bandwidth of 1.07 GHz was generated by the T-shaped resonator, and the higher frequency notched band with a 3-dB bandwidth of 0.61 GHz was realized by etching the L-shaped defect microstrip structure. It can be seen that the first notch band has a fractional bandwidths (FBW) of about 15.47% and the second notch band has a FBW of about 8.76%. Moreover, the measured results show that the dual notched bands are observed at 3.5 and 7.5 GHz with the rejection level of 25.2 dB and 17.3 dB, with a fractional bandwidths (FBW) of 15.91% and 9.63%.

**Figure 8:**
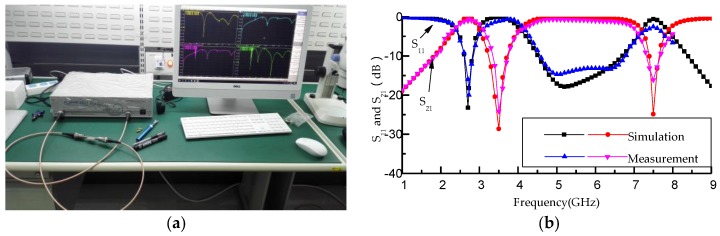
(**a**) The examination photo by Vector Network Analyzer Plannar 804/1 (Copper Mountain Technologies, Indianapolis, IN, USA); (**b**) comparison of the simulation and measurement results of the proposed filter.

Figure 8a shows the examination photo of the fabricated filter by Vector Network Analyzer Plannar 804/1 (Copper Mountain Technologies, Indianapolis, IN, USA) with the frequency range of 8 GHz. The simulation data and the measured data are imported into the Excel form, and the comparison between the simulation results and the measured results is drawn by Origin software (OriginLab Corporation, Northampton, MA, USA) as shown in Figure 8b. According to Figure 8b, the measured insertion loss is lower than 1.2 dB and the reflection coefficient is higher than 15 dB over the ultra-wideband passband. The measured and simulation results have the same resonant frequencies. The equivalent model of resonant elements composed of the T-shaped resonator and L-shaped defect microstrip structure is proven to be correct by the consistent resonant frequencies.

**Figure 9:**
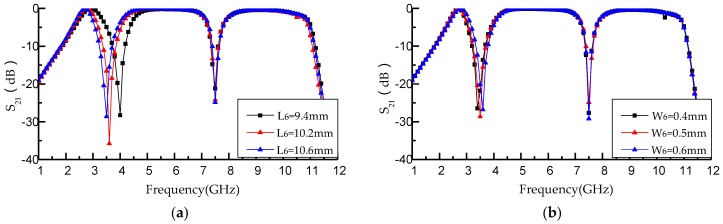
(**a**) Simulation results with different *L*_6_ (*L*_6_ = 9.4 mm, *f*_01_ = 4 GHz, *L*_6_ =1 0.2 mm, *f*_01_ = 3.6 GHz, *L*_6_ = 10.6 mm, *f*_01_ = 3.5 GHz); (**b**) Simulation results with different *W*_6_ (*W*_6_ = 0.4 mm, *f*_01_ = 3.4 GHz, *W*_6_ = 0.5 mm, *f*_01_ = 3.5 GHz, *W*_6_ = 0.6 mm, *f*_01_ = 3.6 GHz).

Figure 9 illustrates the proposed filter performance with different parameters (*g*_1_, *L*_6_, *W*_6_) of the T-shaped resonator. Figure 9a depicts that the proposed filter performance is adjusted by changing L6 of the T-shaped resonator under the condition of *L*_6_ = 2*L*_5_. As *L*_6_ increases from 9.4 to 10.6 mm, *f*_01_ decreases from 4 to 3.5 GHz, while *f*_02_ is still 7.5 GHz. It can be derived from the Equations (4) and (5) that this leads to the decreasing of *f*_01_. Figure 9b describes the proposed filter performance changing with different W_6_ of the T-shaped resonator when *L*_7_ = 11 mm. It can be clearly seen that when *W*_6_ increases from 0.4 to 0.6 mm, *f*_01_ increases from 3.4 to 3.6 GHz, while *f*_02_ is 7.5 GHz. The FBW of the first notched band decreases from 16 to 14.8% and the FBW of the second notched band increases from 11.24% to 10.9%.

**Figure 10:**
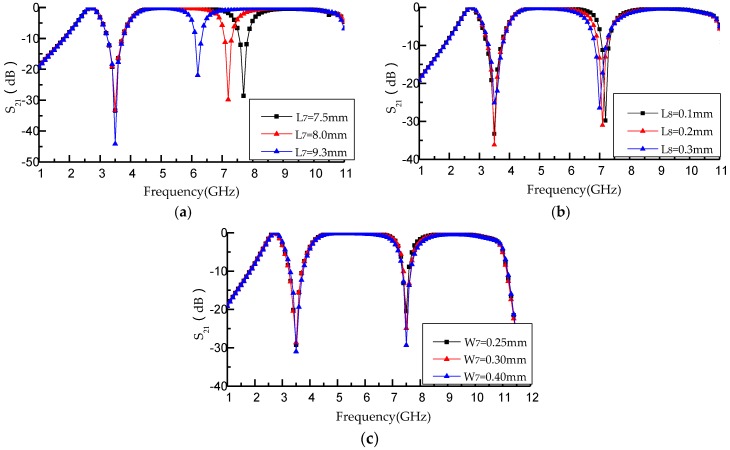
(**a**) Simulation results with different *L*_7_ (*L*_7_ = 7.5 mm, *f*_02_ = 7.7 GHz, *L*_7_ = 8 mm, *f*_02_ = 7.2 GHz, *L*_7_ = 9.3 mm, *f*_02_ = 6.2 GHz); (**b**) simulation results with different *L*_8_ (*L*_8_ = 0.1 mm, *f*_02_ = 7.2 GHz, *L*_8_ = 0.2 mm, *f*_02_ = 7.1 GHz, *L*_8_ = 0.3 mm, *f*_02_ = 7 GHz); (**c**) simulation results with different *W*_7_ (*W*_7_ = 0.2 mm, *W*_7_ = 0.3 mm, *W*_7_ = 0.4 mm, *f*_01_ = 3.5 GHz, *f*_02_ = 7.2 GHz).

Figure 10 respectively illustrates that the performance of the second notched band is adjusted by changing the relevant parameters (*L*_7_, *L*_8_, *W*_7_) of the L-shaped defect microstrip structure. The total length of the defect microstrip structure is equal to *L*_7_ + *L*_8_ and the width is *W*_7_. Figure 10a describes the proposed filter performance with changing *L*_7_ of the L-shaped defect microstrip structure. When *L*_7_ increases from 7.5 to 9.3 mm, *f*_02_ decreases from 7.7 to 6.2 GHz, while *f*_01_ is 3.5 GHz. Figure 10b shows the simulated insertion loss only with different *L*_8_. When *L*_8_ increased from 0.1 to 0.3 mm, *f*_01_ is 3.5 GHz but *f*_02_ decreases from 7.2 to 7 GHz. It can be derived from the equivalent equation of resonant elements that the *C*_2_ of the equivalent circuit increases with the increasing of the total length of the L-shaped defect microstrip structure (*L*_7_ + *L*_8_). Figure 10c illustrates the proposed filter performance with different *W*_7_ of the L-shaped defect microstrip structure. When *W*_7_ increases from 0.2 to 0.4 mm, *f*_01_ and *f*_02_ are maintained at 3.5 GHz and 7.2 GHz. It can be clearly seen that when *W*_7_ increases from 0.2 to 0.4 mm, the FBW of the second notched band increases from 15.71% to 22.68%. The resonant frequency *f* of the L-shaped defect microstrip structure decreases with the increasing of the total length of the L-shaped defect microstrip structure (*L*_7_ + *L*_8_) as shown in Equation (10).

According to the above analysis, the frequency of the second notched band decreases by increasing the total length of the L-Shaped defect microstrip structure (*L*_7_ + *L*_8_) and the fractional bandwidths of the second notched band increases with the increasing of *W*_7_, while the frequency of the first notched band is still at 3.5 GHz.

Finally, a comparison of characteristics between the proposed filter and some other filters is presented in Table 5, in which insertion loss and return loss are over the whole pass-bands, while *f*_L_ and *f*_H_ means the central frequency of lower and higher frequency notched bands. It can be seen that the proposed filter has dual notched bands at 3.5 and 7.5 GHz with the rejection level of 25.2 dB and 17.3 dB and an FBW 123.9% higher than the other 10 works [22,23,24,25,26,27,28,29,30,31]. The measured results illustrate that the dual notched bands are centered at 3.5 and 7.5 GHz with an FBW of 15.91% and 9.63%.

## 4. Conclusions

In this paper, a UWB bandpass filter with dual notched bands using a T-shaped resonator and L-shaped defect microstrip structure is designed and fabricated. The proposed filter has dual adjustable notched bands which are implemented by changing the parameters of the L-shaped defect microstrip structure and T-shaped resonator respectively. As the two simulation results obtained by using ADS and HFSS are consistent, the correctness of the equivalent lumped circuit composed of a T-shaped resonator and L-shaped defect microstrip structure is further verified. The proposed filter can suppress dispensable bands at 3.5 GHz and 7.5 GHz, which can be designed and applied to the wireless communication system at WiMAX band and X-band by adjusting the parameters of the T-shaped resonator and L-shaped defect microstrip structure.

## Figures and Tables

**Table 1:** The circuit parameters in Figure 5b extracted from the advanced design system (ADS).

*C* (pF)	*L* (nH)
*C*_p__1_ = 2.415	*L*_p__1_ = 0.85
*C*_p_ = 2.489	*L*_s1_ = 0.18
*C*_p2_ = 0.1216	*L*_s2_ = −0.105
--	*L*_p2_ = 0.47

**Table 2:** Optimized design parameters of the proposed ultra-wideband filter (The units are mm).

Length	Length	Width	Width	Radius	Gap Length
*L*_1_ = 15	*L*_5_ = 5.3	*W*_1_ = 1.95	*W*_5_ = 0.5	*R*_1_ = 0.15	*g*_1_ = 0.1
*L*_2_ = 5	*L*_6_ = 10.6	*W*_2_ = 3	*W*_6_ = 0.3	*R*_2_ = 0.15	--
*L*_3_ = 4	*L*_7_ = 9	*W*_3_ = 0.3	*W*_7_ = 0.1	--	--
*L*_3_ = 4	*L*_8_ = 0.1	*W*_4_ = 1.4	*W*_8_ = 0.3	--	--

**Table 3:** Simulation results of the dual notched bands.

Notch Frequency (GHz)	3 dB Bandwidth (GHz)	Fractional Bandwidths (FBW) %	Rejection Level (dB)
3.5	1.07	15.62	28.6
7.5	0.61	8.91	25.1

**Table 4:** Measurement results of the dual notched bands.

Notch Frequency (GHz)	3 dB Bandwidth (GHz)	Fractional Bandwidths (FBW) %	Rejection Level (dB)
3.5	1.09	15.91	25.2
7.5	0.66	9.63	17.3

**Table 5:** Comparison of the proposed filter with recently proposed ultra-wideband bandpass filters with notched bands.

Ref.	Insertion Loss (dB)	Return Loss (dB)	*f_L_*(GHz)	*f_H_* (GHz)	Fractional Bandwidths (FBW) %
[22]	0.5	13	5.5	13.6	112
[23]	0.6	12	6.55	8.66	130
[24]	0.65	10	4.9	8.5	110
[25]	1.2	11	4.3	9.1	82.4
[26]	1.1	10	2.4	5.5	110
[27]	1.5	15	2.4	5.2	105
[28]	0.7	15	1.16	3.5	95.5
[29]	1.1	15	2.62	5.32	105
[30]	2	15	5.8	8	92
[31]	1.12	11	2.4	5.2	89.6
This work	0.8	15.5	3.5	7.5	123.9
